# Severe efavirenz associated neurotoxicity: A retrospective cohort study

**DOI:** 10.4102/sajid.v38i1.522

**Published:** 2023-07-24

**Authors:** Priyadarshini Arnab, Roland Croxford, Janet Scott, Sameshan Perumal, Zahraa Mohammed, Lubbe Wiesner, Karen Cohen, Sean Wasserman

**Affiliations:** 1 Department of Medicine, Faculty of Health Sciences, University of Cape Town, Cape Town, South Africa; 2 Department of Health, DP Marais Hospital, Western Cape Government, Cape Town, South Africa; 3 Department of Statistics, Faculty of Sciences, University of Cape Town, Cape Town, South Africa; 4 Division of Clinical Pharmacology, Faculty of Health Sciences, University of Cape Town, Cape Town, South Africa; 5 Division of Clinical Pharmacology, Department of Medicine, Groote Schuur Hospital, University of Cape Town, Cape Town, South Africa

**Keywords:** efavirenz, isoniazid, risk factors, neurotoxicity, cerebellar, Cape Town

## Abstract

**Background:**

Efavirenz (EFV) is associated with neuropsychiatric symptoms. Severe neurotoxicity has been reported but the clinical phenotype and risk factors are poorly defined.

**Objectives:**

To characterise clinical presentations, risk factors and outcomes to help clinicians recognise severe neurotoxicity earlier.

**Method:**

The authors retrospectively identified adults with supratherapeutic EFV concentrations (> 4 mg/L) obtained during routine clinical care in Cape Town, South Africa. Clinical and laboratory data at the time of EFV quantification were extracted from medical records. Logistic regression was performed to identify associations with neuropsychiatric symptoms, and with severe neurotoxicity.

**Results:**

Eighty one patients were included; 62 with neuropsychiatric manifestations (most frequently ataxia [*n* = 20] and psychomotor slowing [*n* = 24]); and 19 with hepatotoxicity. Overall, 28 (34.6%) were male, 49 (60.5%) had concomitant isoniazid exposure, and median EFV concentration was 12.1 mg/L (interquartile range [IQR]: 6.6–20.0). Neuropsychiatric symptoms were associated with longer duration of EFV therapy, adjusted odds ratio (aOR) 1.3/180-day increment (95% confidence interval [CI]: 1.0–1.7); higher EFV concentrations, aOR 1.2/1 mg/L increase (95% CI: 1.0–1.4) and isoniazid exposure, aOR 8.2 (95% CI: 2.5–26.7). Severe neuropsychiatric symptoms occurred in 47 (75%) patients at a median of 5.9 months (IQR: 2.1–40.8) after EFV initiation. Severe symptoms odds were 1.2-fold higher (95% CI: 1.1–1.4) per 1 mg/L increase in EFV concentration. Symptoms resolved completely within 1 month in 25 (76%) patients with severe neurotoxicity who discontinued EFV.

**Conclusion:**

A concentration–effect relationship for severe neurotoxicity exists, which occurred late and resolved in most patients after EFV discontinuation.

**Contribution:**

The authors highlighted clinical heterogeneity and morbidity of EFV-associated neurotoxicity.

## Introduction

Efavirenz (EFV), a non-nucleoside reverse transcription inhibitor, has been a backbone of antiretroviral therapy (ART) for the last 15 years. Daily dosing, inclusion in fixed drug combinations and lack of significant drug–drug interaction with rifampicin have made it a widely used first-line drug in combination with two nucleoside reverse transcriptase inhibitors (NRTIs),^1^ especially in resource-limited countries with high tuberculosis (TB) burdens.^2^

Efavirenz is metabolised in the liver by cytochrome P450 isoenzyme 2B6 (CYP2B6). Single-nucleotide polymorphisms (SNPs) in *CYP2B6*, present in up to 20% of sub-Saharan African populations,^3,4^ confer ‘slow metaboliser’ genotypes and lead to a risk of increased EFV concentrations, particularly in patients on concomitant TB treatment,^5^ as alternate pathways of EFV metabolism are also inhibited by isoniazid (INH).

Two major adverse effects of EFV include hepatotoxicity, secondary to an immunoallergic pathway,^6^ and central nervous system (CNS) toxicity possibly because of direct glial toxicity.^7,8^ Clinical features of CNS EFV toxicity range from sleep and mood disturbances in milder forms^9^ to psychosis, cerebellar ataxia, encephalopathy^10^ and rarely, death^11^; long-term toxicity may promote the development of HIV-associated neurocognitive disorder (HAND).^12^ Apart from host genetics and lower body weight (leading to higher EFV exposures), clinical risk factors for EFV neurotoxicity have not been established. Previous cohort studies have only included women, did not separate severe from milder symptoms and only small case series have documented severe EFV neuropsychiatric sequelae.^10,11,13,14^

High rates of HIV-associated TB place patients in South Africa at particular risk of EFV neurotoxicity, especially given the relatively high background prevalence of ‘slow metaboliser’ genotypes and ongoing use of EFV for ART by some clinicians despite dolutegravir availability. However, the diagnosis may be missed because of overlap with other common neurological syndromes, and pharmacogenetic risk stratification may not be feasible in resource-limited settings. We conducted a retrospective cohort study to describe the clinical phenotype of severe EFV-induced neurotoxicity and explore risk factors for its development.

## Methods

### Study population

We searched the University of Cape Town Clinical Pharmacology laboratory database for EFV concentrations measured in routine care at five public sector Cape Town hospitals between February 2008, when the current database was started, and July 2017; monitoring of EFV concentrations is conducted for clinical suspicion of toxicity or for adherence checks. Medical records of patients with elevated concentrations > 4 mg/L (normal range 1 mg/L – 4 mg/L) were retrieved and reviewed. We included data from all patients over the age of 18 years with available records.

### Clinical data

The following data were extracted from medical records, national laboratory services and picture archiving and communications system: biometrics including age, weight, and gender; treatment history relating to ART, TB therapy and isoniazid preventive therapy (IPT) and clinical manifestations at the time of, and subsequent to, EFV toxicity. Results of blood, cerebrospinal fluid (CSF) and radiological investigations were recorded to exclude other causes of neuropsychiatric syndromes including: neurosyphilis; bacterial, TB or, cryptococcal meningitis; neurological TB immune reconstitution inflammatory syndrome (IRIS); stroke and electrolyte abnormalities and hypoglycaemia. Data were captured using unique participant identifiers onto paper case report forms and entered into an electronic database (REDCap).

Efavirenz-associated neurotoxicity was defined as the presence of known neuropsychiatric manifestations of EFV toxicity without an alternative clinical or radiological explanation. Indicative clinical features included ataxia or cerebellar signs, psychomotor slowing (including slowed speech, decreased movement, impaired cognitive function and catatonia), mood disorders, psychosis, sleep disorders and confusion.^9,12,13,15^ Severe EFV-associated neurotoxicity was defined as a Division of Allergy and Infectious Diseases (DAIDS) altered mental status Grade 3 or more (Confusion, memory impairment, lethargy and somnolence causing inability to perform usual social and functional activities; or delirium, obtundation or coma),^16^ and/or ataxia of DAIDS Grade 3 or more. A pre-specified case definition of EFV toxicity was applied to information extracted from clinical records. Cases were then classified according to DAIDS criteria. Patients fulfilling eligibility criteria (which included having supratherapeutic EFV concentrations) but no neuropsychiatric effects were included as ‘non-neuropsychiatric’ cases.

### Analysis

Descriptive statistics were used to summarise the demographic and clinical characteristics of the study population. Univariable logistic regression was performed to determine associations between pre-specified predictors and the primary outcome of severe EFV-associated neurotoxicity. Independent variables included age, weight, EFV concentration, duration of EFV therapy, isoniazid exposure (either as TB therapy or IPT) and gender. Data completeness were used to determine a candidate set of variables for inclusion in a multivariable model with only variables with < 20% missing data included. This set was reduced by a backward step-wise model elimination using the Akaike Information Criterion (AIC) as the optimising criteria. We also used logistic regression to explore factors associated with the presence of neuropsychiatric symptoms of any severity, using cases with hepatitis (and no neurological manifestations) as a comparator. We checked for multicollinearity by testing the correlation between clinically linked variables and quantifying effects on model parameters – predictors that resulted in increased variance greater than or equal to 10% without an impact on coefficient size were dropped from the final model to avoid collinearity. The Hosmer–Lemeshow statistic was used to assess the calibration of the final model; discriminative ability was quantified by the area under the receiver operating characteristic (ROC) curve. Survival was represented using Kaplan–Meier plots with censoring at 3 months after initial supratherapeutic EFV concentration. Time to development of EFV toxicity after ART initiation was represented as an empirical cumulative distribution function, stratified by severity. Statistical analysis was conducted using R software, version 3.6.1.

### Ethical considerations

Ethical approval for this study was obtained from Human Ethics Research Committee of the University of Cape Town (843/2016).

## Results

### Clinical phenotype and outcomes

A total of 109 patients with supratherapeutic EFV concentrations were identified over the study period; data from 81 patients were included in the analysis, distributed as follows: Brooklyn Chest Hospital (*n* = 1), DP Marais (*n* = 16), Groote Schuur Hospital (*n* = 54), Mitchell’s Plain District Hospital (*n* = 8) and New Somerset Hospital (*n* = 2) (See consort diagram Appendix 1, Figure 1-A1). Patients were excluded if their clinical records were missing or if they were under the age of 18 years at the time of EFV sampling. One patient had chronic diarrhoea and no other apparent indication of having had an EFV concentration performed and was also excluded. All patients were HIV-positive and were prescribed EFV as part of their ART.

A total of 62 patients had a neuropsychiatric syndrome and 19 had hepatitis as a reason for EFV sampling. Overall, 28 (34.6%) patients were male and the median age was 37.5 years (interquartile range [IQR]: 29.3–45.0). Median CD4 count was 261 cells/mm^3^ (IQR: 101–412); 41 (74.6%) had undetectable plasma HIV RNA.

Compared to patients with hepatitis (non-neuropsychiatric), those with neuropsychiatric manifestations had a lower median weight (50 kg vs 71 kg), lower median CD4 (195 cells/µL vs 448 cells/µL) and higher EFV concentrations (16.1 mg/L vs 6.6 mg/L); a higher proportion of patients with neuropsychiatric presentations were exposed to INH (44 [74.6%] vs 5 [26.3%] with hepatitis) (Table 1). All cases with hepatotoxicity were reviewed by a hepatologist, including liver biopsy in many cases, and concluded to have EFV-induced liver injury.

**TABLE 1:** Clinical characteristics at time of supratherapeutic efavirenz concentration.

Variable	Neuropsychiatric (*n* = 62)					Hepatitis (*n* = 19)					*p*
	*n*	%	Median	IQR	Range	*n*	%	Median	IQR	Range	
Male gender (*n* = 81)	28	45.2	-	-	-	0	-	-	-	-	< 0.01
Weight (kg) (*n* = 63)	-	-	50	42.1–56.5	-	-	-	71	65.5–82.5	-	< 0.01
Age at toxicity (years) (*n* = 81)	-	-	39.1	30.9–46.1	-	-	-	32.8	27.7–38.5	-	0.12
CD4 (cells/µL) (*n* = 81)	-	-	195	74–320	-	-	-	449	320–517	-	< 0.01
EFV concentration (mg/L) (*n* = 81)	-	-	16.1	7.5–20.0	4–20	-	-	6.6	4.9–8.8	4–16	< 0.01
HIV RNA copies/mL < 40 (*n* = 55)	30	71.4	-	-	-	11	100	-	-	-	0.05
Duration of EFV therapy (months) (*n* = 74)	-	-	5.9	2.1–40.8	0.7–113	-	-	5.9	2.6–10.4	0.4–17.2	0.27
Exposure to INH (*n* = 78) TB treatment INH prophylaxis	44 33 11	74.6 55.9 18.7	- - -	- - -	- - -	5 3 2	26.3 15.8 10.5	- - -	- - -	- - -	< 0.01 0.003 0.50
Duration of TB therapy or IPT (days) (*n* = 79)	-	-	48	30.0–108.2	7–554	-	-	30	26.8–38.2	17–63	0.19
Laboratory parameters† Hb (g/dL) (*n* = 81) Cr (μmol/L) (*n* = 80) ALT (U/L) (*n* = 75)	- - -	- - -	11.8 59 32	9.5–13.5 47.5–70.5 20–54.5	- - -	- - -	- - -	13.5 51 665	11.8–14.2 46.5–62.5 274–1353	- - -	0.03 0.12 < 0.01

Note: Continuous variables compared using Wilcoxon rank sum and categorical variables using Fishers test.

EFV, efavirenz; HIV, human immunodeficiency virus; DAIDS, Division of Allergy and Infectious Diseases; INH, isoniazid; TB, tuberculosis; IPT, isoniazid preventative therapy; Hb, haemoglobin; Cr, creatinine; ALT, alanine transaminase; IQR, interquartile range; U/L, unit per litre.

† , Investigations performed within one month of index presentation.

Of those with available results, 7 out of 44 (15.9%) patients with neuropsychiatric symptoms had abnormal CSF findings at the time of index presentation, six with raised protein (> 0.45 g/dL) and variable pleocytosis and one with isolated polymorphonuclear pleocytosis. Neuroimaging (either computed tomography [CT] or magnetic resonance imaging [MRI]) was conducted for patients with neuropsychiatric symptoms, with 18 being reported as abnormal. Abnormalities included ring enhancing lesions (*n* = 4), generalised atrophy (*n* = 5), infarcts (*n* = 3) and non-specific white matter changes (*n* = 3). Fourteen patients had positive serum treponemal tests, but only one had a positive rapid plasma reagent (RPR), titre 1:1.

Psychomotor slowing (*n* = 24) was the most common neuropsychiatric symptom, followed by ataxia (*n* = 20), psychosis (*n* = 17), other cerebellar signs (*n* = 13) and mood disturbances (*n* = 11). Division of Allergy and Infectious Diseases Grade 3 or higher symptoms for ataxia, altered mental state and psychiatric disorders was present in 47 (75.8%) patients. Median time to neurotoxicity was 5.9 months (IQR: 2.1–40.8); those with milder manifestations presented later (9.8 months [IQR: 1.6–19.0]) compared with patients with severe symptoms (5.7 months [IQ: R 2.7–42.3]), although this difference was not statistically significant (*p* = 0.73) (Figure 1). Median EFV concentration was 20.0 mg/L (the upper limit of assay detection, IQR: 13.0–20.0) in those with severe versus 7.0 mg/L (IQR: 4.5–11.8) in those with mild neurotoxicity (*p* < 0.01).

**FIGURE 1:**
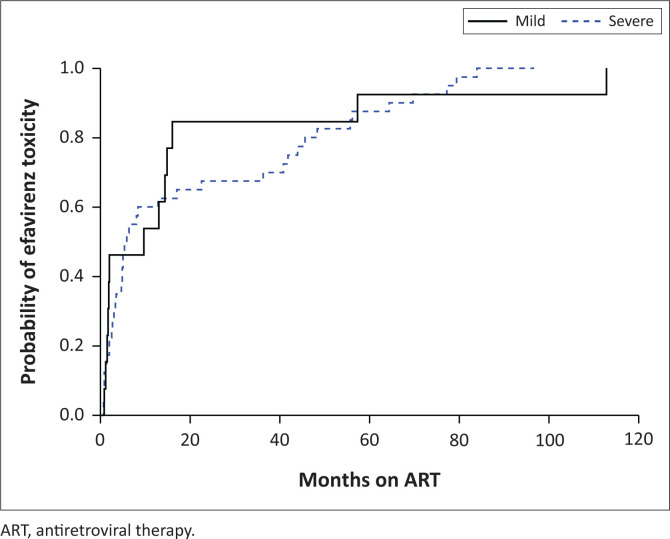
Empiric cumulative distribution function showing probability of efavirenz toxicity over time.

At the time of EFV concentration sampling, 12 (19%) patients with neuropsychiatric symptoms had been noted in their clinical records as having suspected HAND and 6 (10%) were documented to have depression.

Efavirenz was stopped in all those for whom supratherapeutic concentrations were detected. A total of 34 patients were started on a protease inhibitor (PI) regimen and 18 were changed to nevirapine. Only one patient was re-challenged with EFV.

Fourteen patients in the hepatitis group (74%) and 46 (74%) of those with neuropsychiatric presentations improved on withdrawal of EFV; the condition remained unchanged at 1 month in 6 (8%) patients. Among those with severe neurotoxicity, there was complete resolution of symptoms within 1 month in 25 out of 33 (76%) who are recorded to have discontinued EFV.

Fourteen (17%) patients died within the 3-month follow-up period, nine (64%) were associated with neuropsychiatric presentations, five (55.6%) of which were categorised as severe. Four patients had drug-resistant TB, two patients had sepsis, and three had generalised seizures. Five (36%) patients without neuropsychiatric symptoms died in hospital, all secondary to fulminant liver failure, two of whom were less than 3 months post-partum. Median time to death after diagnosis of EFV toxicity was 21 days (IQR: 10.0–36.0) overall; 20 days (IQR: 9.0–42.3) for neuropsychiatric presentations and 22 days (IQR: 12.5–35.5) for non-neurological presentations (Figure 2).

**FIGURE 2:**
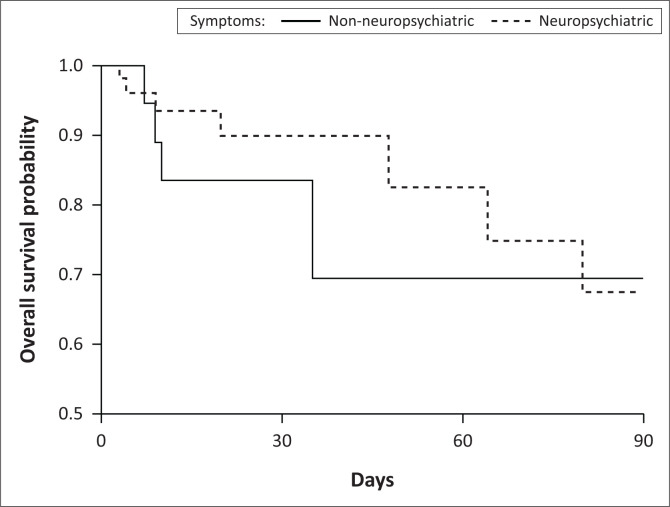
Kaplan–Meier plot comparing survival probability of neuropsychiatric and non-neuropsychiatric (hepatitis) symptoms.

### Predictors of neurotoxicity

Neuropsychiatric symptoms were associated with longer duration of EFV therapy, aOR 1.3 per 180-day increment (95% confidence interval [CI]: 1.0–1.7); higher EFV concentrations, aOR 1.2 per 1 mg/L increase (95% CI: 1.0–1.4) and isoniazid exposure, aOR 8.2 (95% CI: 2.5–26.7) (Table 2). An increase in the CD4 count (aOR 0.8 per 50 cells/µL; 95% CI: 0.7–0.9) and an increase in weight (aOR 0.3 per 10 kg; 95% CI: 0.1–0.5) were protective against the development of neuropsychiatric symptoms. We could not explore if there was a relationship between sex and neuropsychiatric effects as the hepatitis group were all female.

**TABLE 2:** Associations with neuropsychiatric symptoms.

Variable	Univariable		*p*	Multivariable		*p*
	OR	95% CI		OR	95% CI	
Age	1.0	1.0–1.1	0.29	†	-	†
Weight (per 10 kg increase)	0.3	0.1–0.5	< 0.01	†	-	†
EFV concentration (per 1 mg/L increase)	1.2	1.1–1.4	< 0.01	1.3	1.1–1.6	0.01
Duration of EFV therapy (per 180 day increase)	1.3	1.0–1.7	0.06	1.99	1.1–3.7	0.03
Duration of TB therapy (weeks)	1.2	0.9–1.6	0.24	†	-	†
INH exposure	8.2	2.5–26.7	< 0.01	†	-	†
CD4 cell count, per 50 (cells/µL)	0.8	0.7–0.9	< 0.01	0.70	0.6–0.9	< 0.01

EFV, efavirenz; INH, isoniazid; TB, tuberculosis; OR, odds ratio; 95% CI, 95% confidence interval; AIC, Akaike Information Criterion.

† , Backward elimination using AIC = (54.5→52.5) Chi-squared: 3.49; Hosmer-Lemmeshow *p* = 0.89, g = 10 (*n* = 69 for multivariable model).

Higher EFV concentrations were associated with severe neuropsychiatric symptoms, with 1.2-fold higher odds (95% CI: 1.1–1.4) with every 1 mg/L increase and 3.1-fold higher odds (95% CI: 1.4–6.8) for every 5 mg/L increase (Table 3).

**TABLE 3:** Associations with severe neuropsychiatric symptoms.

Variable	Univariable		*p*	Multivariable		*p*
	OR	95% CI		OR	95% CI	
Age	1.0	0.9–1.0	0.34	†	-	†
Weight (10 kg)	0.6	0.3–1.2	0.18	†	-	†
EFV concentration (per 1 mg/L increase)	1.2	1.1–1.4	< 0.01	1.3	1.1–1.5	< 0.01
Duration of EFV therapy (per 180 days)	1.0	0.9–1.2	0.73	0.9	0.8–1.1	0.30
Duration of TB therapy (weeks)	0.9	0.9–1.0	0.10	†	-	†
INH exposure	2.3	0.6–9.5	0.26	†	-	†
CD4 cell count, per 50 (cells/µL)	1.0	0.8–1.2	0.93	†	-	†
Male gender	0.4	0.1–1.5	0.17	†	-	†

EFV, efavirenz; INH, Isoniazid; TB, tuberculosis; OR, odds ratio; 95% CI, 95% confidence interval; AIC, Akaike Information Criterion.

† , Backward elimination using AIC = (47.0→39.3); Chi-squared: 4.9; Hosmer-Lemmeshow: *p* = 0.76, *g* = 10 (*n* = 47 for multivariable model).

## Discussion

We describe the clinical phenotype and factors that may contribute to higher risk of neurotoxicity among 81 patients with elevated EFV concentrations. There were duration- and concentration-dependent effects, as well as higher risk with concomitant INH exposure and those with lower CD4 count. Most patients with severe neurotoxicity had symptom resolution within 1 month after stopping EFV, although overall 3-month mortality was high in this population.

Prior adult studies involved smaller case series of neuropsychiatric symptoms in female patients.^13,14^ Our study is one of the few to include men in the neuropsychiatric cohort. Female gender has been associated with higher EFV concentrations compared with men, possibly because of a higher dose for weight with standard fixed drug combinations.^17,18^ However, more recent epidemiological data show a shift towards higher body weight in women in South Africa, potentially resulting in lower EFV concentrations.^19^ Men did have a higher median EFV concentration (15.7 mg/L; IQR: 6.9–20 vs 10.8 mg/L in women; IQR: 6.6–20, *p* = 0.27, supplementary Figure 2-A1), but we could not ascertain weights from many of the records, so we cannot conclude this difference is because of weight alone.

The protective effect of a higher CD4 count against neuropsychiatric symptoms may also be linked to weight – lower CD4s are associated with lower weights.^20^ Lower CD4 counts are also associated with higher risk of TB,^3^ placing patients at a higher chance of being exposed to INH, either as part of TB treatment or prophylaxis, which would also increase their risk of EFV toxicity and neuropsychiatric symptoms.

The association between longer duration of EFV therapy and neuropsychiatric symptoms may be linked to direct toxicity of glial cells from EFV metabolites,^6^ with a cumulative exposure threshold necessary for symptom appearance. Although the exact mechanism of EFV hepatitis symptoms remains unclear, suggested mechanisms include an EFV-induced mitochondrial dysfunction pathway.^21^ This potential difference in toxicity mechanism may explain why patients with hepatitis appeared to have poorer survival outcomes in the earlier part of their illness, with fulminant liver failure being the cause of death, although the difference was not statistically different (*p* = 0.38). Decreased survival from neurotoxicity occurred later, coinciding with the later onset of toxicity with prolonged course. A higher EFV concentration was associated with neuropsychiatric but not non-neuropsychiatric symptoms, supporting the hypothesis that there may be different pathological mechanisms in the clinical manifestations of EFV toxicity.

Higher EFV concentration was the only independent risk factor for severe neuropsychiatric symptoms. Symptoms of neuropsychiatric toxicity can be vague and non-specific. Patients may have presented later as they only sought medical assistance once symptoms were severe enough to impede daily functioning, and similarly, clinicians may have only requested EFV concentrations when they thought symptoms were serious enough to change to alternative ART. The median time to EFV neurotoxicity was 6 months in the current study, but other case series have noted much longer delays of up to 2 years,^14^ which might reflect a greater awareness of the presentation of EFV toxicity during our study, as the lack of clinician awareness was cited previously as a reason for delayed diagnosis.^14^

Consistent with other case reports, cerebellar signs and neuropsychiatric symptoms often co-exist or pre-date each other.^13,14^ Nineteen per cent of patients with a pre-existing diagnosis of suspected HIV-associated encephalopathy is also in keeping with the finding of long-term neurocognitive depression associated with EFV toxicity.^15,22^

Inhibited by Isoniazid is a known risk factor for increased EFV toxicity in those with SNPs^23,24^ for slow metaboliser genotypes, and in our population INH exposure was a significant risk factor for the development of EFV toxicity and neuropsychiatric symptoms. Pharmacokinetic and pharmacogenetic studies have shown that clearance of INH in patients taking both EFV and INH is highly dependent on the *NAT2* or *CYP2B6* polymorphisms, with presence of the *NAT2* and *CYP2B6* mutations having as much as a five-fold difference in EFV clearance between ‘slow’ and ‘normal’ metabolisers.^23^ With an estimated 30% – 52.5% of South Africa’s population having a slow *NAT2* mutation,^25^ and our data suggesting concentration-dependent toxicity, genotyping should ideally be performed prior to co-administering EFV and INH is known to be a risk.^26^ The association with INH co-administration may also explain why some patients also have a prolonged asymptomatic period – patients became toxic once INH was co-administered and symptoms took a month to resolve after EFV had been stopped.

Inability to obtain accurate, complete data from medical records is a limitation of all retrospective studies. Pre-specified predictors, such as weight, were excluded from multivariable models because of missing data, potentially influencing outputs. Statistical analysis was also further complicated by certain subsets within the cohort being much larger than others, which also likely influenced the outputs of both univariate and multivariate models. Clinical notes detailing the neuropsychiatric condition of patients were not recorded in a standardised manner so that symptom severity may have been misclassified. Interobserver variation was, however, limited by one researcher collecting all the data. Although patients with neuropsychiatric symptoms had CSF and neuroradiological abnormalities, on reviewing the totality of the clinical information, we are fairly certain (acknowledging the limitations of retrospective analysis) that EFV was an important contributor. We excluded other conditions to the best of our ability, but as the patient cohort was hospitalised inpatients with multiple co-existing comorbidities, an undiagnosed or untreated contributor cannot be excluded. Potential cases with neurotoxicity but within the therapeutic EFV concentration range would not have been identified within this cohort, but the finding of a dose-dependent relationship suggests that supratherapeutic concentrations are more likely to lead to neuropsychiatric symptoms.

Ethnicity, a surrogate for metaboliser phenotype, was not included as a parameter in this study as this variable was not consistently recorded and could not be inferred from the available demographic information in the hospital records. We were unable to collect samples for genotyping because of difficulties contacting patients.

## Conclusion

This study highlights the clinical heterogeneity of EFV-associated neurotoxicity. Efavirenz toxicity is a reversible condition and recognition is critical to avoid misdiagnosis with potentially fatal outcome with continuation of EFV. Our findings support replacement of EFV by the integrase inhibitor dolutegravir as a first-line drug in ART programmes.^27,28^

## Figures and Tables

**FIGURE 1-A1:**
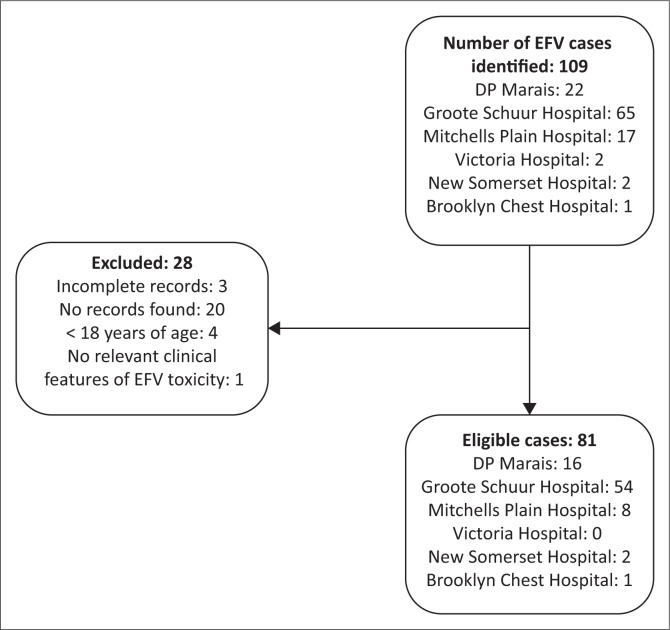
Consort diagram showing selection of cases for study.

**FIGURE 2-A1:**
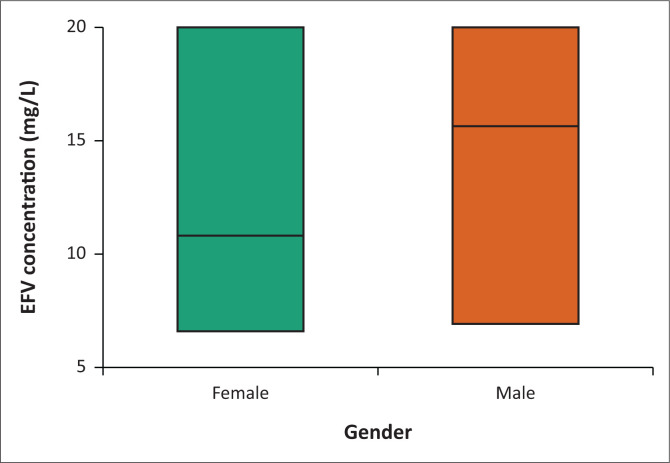
Box and whisker plot showing distribution of Efavirenz concentrations by gender.
